# Attrition from antiretroviral treatment services among pregnant and non-pregnant patients following adoption of Option B+ in Haiti

**DOI:** 10.1080/16549716.2017.1330915

**Published:** 2017-06-22

**Authors:** Jean Wysler Domercant, Nancy Puttkammer, Paul Young, Krista Yuhas, Kesner François, Reynold Grand’Pierre, David Lowrance, Michelle Adler

**Affiliations:** ^a^ Division of Global HIV & TB, Centers for Disease Control and Prevention, Port au Prince, Haiti; ^b^ International Training and Education Center for Health, University of Washington, Seattle, WA, USA; ^c^ Division of Global HIV/AIDS, Centers for Disease Control and Prevention, Atlanta, GA, USA; ^d^ Center for AIDS Research, University of Washington, Seattle, WA, USA; ^e^ National AIDS Control Program, Ministry of Health of the Government of Haiti; ^f^ Department of Family Health, Ministry of Health of the Government of Haiti; ^g^ Division of Global HIV & TB, Centers for Disease Control and Prevention, Kampala, Uganda

**Keywords:** HIV, attrition, ART, Option B+

## Abstract

**Background**: Access to antiretroviral therapy (ART) has expanded in Haiti because of the adoption of Option B+ and the revision of treatment guidelines. Retention in care and treatment varies greatly and few studies have examined retention rates, particularly among women enrolled in Option B+.

**Objective**: To assess attrition among pregnant and non-pregnant patients initiating ART following adoption of Option B+ in Haiti.

**Methods**: Longitudinal data of adult patients initiated on ART from October 2012 through August 2014 at 73 health facilities across Haiti were analyzed using a survival analysis framework to determine levels of attrition. The Kaplan–Meier method and Cox proportional hazards regression were used to examine risk factors associated with attrition.

**Results**: Among 17,059 patients who initiated ART, 7627 (44.7%) were non-pregnant women, 5899 (34.6%) were men, and 3533 (20.7%) were Option B+ clients. Attrition from the ART program was 36.7% at 12 months (95% CI: 35.9–37.5%). Option B+ patients had the highest level of attrition at 50.4% at 12 months (95% CI: 48.6–52.3%). While early HIV disease stage at ART initiation was protective among non-pregnant women and men, it was a strong risk factor among Option B+ clients. In adjusted analyses, key protective factors were older age (*p* < 0.0001), living near the health facility (*p* = 0.04), having another known HIV-positive household member (*p* < 0.0001), having greater body mass index (BMI) (*p* < 0.0001), pre-ART counseling (*p* < 0.0001), and Cotrimoxazole prophylaxis during baseline (*p* < 0.01). Higher attrition was associated with rapidly starting ART after enrollment (*p* < 0.0001), anemia (*p* < 0.0001), and regimen tenofovir+lamivudine+nevirapine (TDF+3TC+NVP) (*p* < 0.001).

**Conclusions**: ART attrition in Haiti is high among adults, especially among Option B+ patients. Identifying newly initiated patients most at risk for attrition and providing appropriate interventions could help reduce ART attrition.

## Background

Global expansion of access to HIV antiretroviral therapy (ART) has exceeded targets, with over 17 million patients receiving ART including 10.3 million in eastern and southern Africa and 1.1 million in Latin America and the Caribbean [1]. The new World Health Organization (WHO) treatment guidelines in response to the Strategic Timing of Antiretroviral Treatment (START) trial findings recommend universal access to treatment for all patients diagnosed with HIV to further accelerate expansion of access to treatment [2,3].

Similarly, in Haiti, with the support of the President’s Emergency Plan for AIDS Relief (PEPFAR) and The Global Fund for AIDS, TB and Malaria, coverage of ART services has greatly expanded since 2003. The adoption of the Option B+ policy which offers lifelong ART to all HIV-infected pregnant and breastfeeding women [4,5] and the revision of the treatment guidelines greatly contributed to that expansion. More than 80,000 patients are receiving ART including nearly 90% of HIV-infected pregnant and breastfeeding women [6].

Retention in care and treatment is an essential determinant of HIV treatment success, including viral load suppression [7,8]. Since the early phases of ART scale-up in low- and middle-income countries (LMICs), support for adherence and retention in care has been an important component of treatment programs. Early studies on ART retention have shown promising results, with many national and sub-national programs achieving average retention levels of ≥ 75% at 12 months after initiation [9–11]; however, retention rates vary considerably [8,12].

From their inception, Haitian ART services have incorporated patient *accompagnateurs* by lay cadres and peer support staff to promote retention [12,13]. Early reports from both urban and rural health facilities demonstrate moderate to high levels of sub-national retention [10,12,13]. However, data from other LMICs have shown that as ART programs mature, and the number of people initiating ART increases over time, incidence of loss to follow-up (LTFU) may increase as well [8]. Over three years after the 2012 adoption and roll-out of Option B+ in Haiti and the implementation of the 2013 WHO guidelines on adult HIV treatment, few studies have examined attrition among patients initiating ART or explored attrition among pregnant women initiating ART [14–16]. The objective of the analysis presented here is to determine the level of attrition in a large cohort of pregnant and non-pregnant adults initiating ART from October 2012 through August 2014 from 73 health facilities in Haiti: We also assess the risk factors associated with ART attrition notably in the context of the nationwide rollout of ‘test and start’.

## Methods

### Data sources

Haiti has three electronic medical record (EMR) systems serving nearly all patients enrolled in HIV care and treatment services. Two of the EMR support specific clinical networks while the third and largest EMR, known as *iSanté*, is operated by the Ministry of Public Health and Population (MOH) and used at nearly 90% of health facilities providing HIV care and treatment services representing 67% of active ART patients. Longitudinal patient-level data from all three EMRs are merged within the national HIV/AIDS surveillance system (HASS). For the purposes of this study, we used routinely available data from iSanté.

Patient data collected at each health facility using iSanté are securely transferred daily to a central server located at the MOH in Port-au-Prince, or as Internet connectivity permits. The study used de-identified data from the iSanté central server. HIV program staff are able to denote official discontinuation of patients from the HIV care and treatment program at their facility due to death, patient or provider preference, or transfer to another health facility (known transfers). Staff use routinely collected data from iSanté, and laboratory and pharmacy registers to evaluate LTFU, based upon the MOH definition of being > 90 days late for a scheduled appointment. Via the iSanté central server database, a patient-matching algorithm was applied to identify duplicate patient records; in instances where duplicate records were identified for the same patient, the first record documenting ART use was used as the index record. To estimate the level of unofficial transfer to other ART sites within Haiti’s health system or ‘silent transfer’ [17], the records of iSanté-based attrition cases were re-identified and matched within the HASS system. Study investigators were not involved in the matching process, since handling identified patient data was not covered by this study’s institutional review board (IRB) clearance. For maximal privacy protection, only aggregate counts of silent transfers among suspected LTFU ART patients were provided to investigators.

### Study sites and population

The study used routinely collected data from patients newly enrolled on ART from October 2012 through August 2014 at 73 health facilities using iSanté, representing 69% of those using iSanté nationwide (see Supplemental Digital Content 1: Study Population). Patient data from 32 health facilities were excluded when the iSanté central server was out-of-date or incomplete, defined as less than 90% of patient encounter data saved to iSanté within 90 days of an appointment, whether clinical or for pharmacy refill, or when the health facility was an ART clinic in a prison (N = 3). Patient records with missing age or sex, duplicate identifiers, and fewer than 90 days of follow-up time possible prior to the administrative close date of the study were excluded from the analysis.

### Measurements

The outcome of interest was all-cause attrition from the ART program. Patients who missed an expected ART refill visit and did not show up at the clinic for at least 90 days afterwards were counted as cases of attrition. The attrition date was the date 90 days after the missed ART refill visit, or the date of an officially recorded discontinuation due to death or patient/health care provider preference, whichever came first. For the purposes of this analysis, patients who were officially transferred out (as noted on an official discontinuation form) and active on ART elsewhere were not categorized as among the patients experiencing attrition from ART.

New ART patients were classified into 3 groups: non-pregnant/non-breastfeeding women, Option B+ clients (any women who initiated ART during pregnancy or within 12 weeks postpartum regardless of CD4 count or WHO stage), and men. The iSanté database contains information on pregnancy and labor and delivery, but not on breastfeeding status. A 12-week postpartum time window was used as a proxy to identify women who were eligible for Option B+ based upon lactation in accordance with the demographic health survey (2012) showing over 95% breastfeeding during that period. Pregnancy start and end dates were estimated using labor and delivery data and data on last menstrual period (LMP) in iSanté. When either LMP or delivery date was missing, then a typical pregnancy duration of 280 days was assumed. In instances where records indicated a pregnancy-related diagnosis or when prevention of mother to child transmission (PMTCT) was listed as a reason for ART eligibility, but no LMP or delivery date was available, we imputed pregnancy start and end dates by assuming that such events occurred at the midpoint of a typical pregnancy of 280 days’ duration. There were 12 patient records where sex was marked as male, but pregnancy-related data were noted for the person. These were assumed to be instances of data entry error leading to misclassification of the sex variable, and were reclassified as female patients initiating ART under Option B+.

Patient demographic covariates were measured at HIV care and treatment registration. Clinical and laboratory covariates were measured in the 90 days prior to ART initiation, with the exception of body mass index (BMI), which was measured within the 180 days prior to ART initiation. Weight measures falling after the first trimester of pregnancy were not used to calculate baseline BMI, since pregnancy-related weight gain could distort a patient’s BMI as a marker of nutritional status and health. A combined variable for HIV disease stage, based upon WHO clinical staging or baseline CD4, whichever indicated more advanced disease, was created (labeled in our results as ‘WHO stage’).

### Data analysis

For our primary analysis, we used survival analysis to estimate time from ART initiation to attrition. We considered only time to first attrition for patients who met attrition criteria more than once. We used health facility-specific censoring dates that reflected the recency of the extracted data. We treated known transfers as censored as of the transfer date. We used the Kaplan–Meier method to describe attrition by patient group and by other covariates, and the Cox proportional hazards regression method to identify factors associated with attrition [18]. The Cox models were stratified by health facility to account for correlation among patients seen at the same facility. We ran separate Cox regression models for each of the three patient groups, and examined whether there were meaningfully different patterns of association across the groups. The authors’ judgement of the results by group guided selection and testing of an interaction term for patient group and WHO stage within the final model. For the duration of pre-ART care, we examined attrition risk for decile groups of the continuous covariate, and created a binary variable at the cut point where a large increase in risk was observed (7 days).

Missing data were present in several covariates (Table 1). Approximately 20% of cases were complete in all covariates. We used multiple imputation by chained equations to impute missing covariate data for the Cox models. We specified 80 imputations, following the guideline that the number of imputations be equal to the proportion of incomplete cases [19]. Multiple imputation assumes that patterns in missing data can be explained by the observed covariates, an untestable assumption. We assessed for violations in the assumption of proportional hazards using Schoenfeld residuals.

**Table 1.** Patient characteristics, by group.

	All patients (n, %)	Option B+ (n, %)	Non-pregnant women (n, %)	Men (n, %)
	17,059	3533	7627	5899
Marital status*				
Married/partnered	8778 (51.5)	2348 (66.5)	3478 (45.6)	2952 (50.0)
Widowed/divorced	2205 (12.9)	153 (4.3)	1424 (18.7)	628 (10.6)
Single	2966 (17.4)	415 (11.7)	1310 (17.2)	1241 (21.0)
Missing/unknown	3110 (18.2)	617 (17.5)	1415 (18.6)	1078 (18.3)
Age group*				
< 25 years	2267 (13.3)	1115 (31.6)	808 (10.6)	344 (5.8)
25–35 years	5787 (33.9)	1784 (50.5)	2369 (31.1)	1634 (27.7)
35–50 years	6238 (36.6)	619 (17.5)	3037 (39.8)	2582 (43.8)
> 50 years	2767 (16.2)	15 (0.4)	1413 (18.5)	1339 (22.7)
Location of residence relative to clinic*				
Different commune	6376 (37.4)	1042 (29.5)	2899 (38.0)	2435 (41.3)
Same commune	10,348 (60.7)	2420 (68.5)	4570 (59.9)	3358 (56.9)
Missing/unknown	335 (2.0)	71 (2.0)	158 (2.1)	106 (1.8)
Household size				
1–3 members	5591 (32.8)	1165 (33.0)	2532 (33.2)	1894 (32.1)
4+ members	1840 (10.8)	357 (10.1)	867 (11.4)	616 (10.4)
Missing/unknown	9628 (56.4)	2011 (56.9)	4228 (55.4)	3389 (57.5)
Other known HIV+ person in household*				
No	6244 (36.6)	1336 (37.8)	2865 (37.6)	2043 (34.6)
Yes	1187 (7.0)	186 (5.3)	534 (7.0)	467 (7.9)
Missing/unknown	9628 (56.4)	2011 (56.9)	4228 (55.4)	3389 (57.5)
Timing of ART start*				
Oct12–Mar13	4233 (24.8)	942 (26.7)	1893 (24.8)	1398 (23.7)
Apr13–Sep13	5583 (32.7)	1225 (34.7)	2466 (32.3)	1892 (32.1)
Oct13–Mar14	4386 (25.7)	913 (25.8)	1883 (24.7)	1590 (27.0)
Apr14–Sep14	2857 (16.7)	453 (12.8)	1385 (18.2)	1019 (17.3)
Starting ART regimen*				
TDF-3TC-EFV	12,684 (74.4)	3002 (85.0)	5269 (69.1)	4413 (74.8)
AZT-3TC-EFV	2376 (13.9)	243 (6.9)	940 (12.3)	1193 (20.2)
AZT-3TC-NVP	1230 (7.2)	217 (6.1)	869 (11.4)	144 (2.4)
TDF-3TC-NVP	525 (3.1)	40 (1.1)	422 (5.5)	63 (1.1)
All other	244 (1.4)	31 (0.9)	127 (1.7)	86 (1.5)
ART start within 7 days of enrollment*				
No	12,702 (74.5)	1356 (38.4)	6400 (83.9)	4946 (83.8)
Yes	4357 (25.5)	2177 (61.6)	1227 (16.1)	953 (16.2)
WHO stage at baseline (by staging or CD4)*				
I	2782 (16.3)	1549 (43.8)	807 (10.6)	426 (7.2)
II	2804 (16.4)	839 (23.7)	1195 (15.7)	770 (13.1)
III	6207 (36.4)	576 (16.3)	3179 (41.7)	2452 (41.6)
IV	4265 (25.0)	162 (4.6)	2064 (27.1)	2039 (34.6)
Missing/unknown	1001 (5.9)	407 (11.5)	382 (5.0)	212 (3.6)
Baseline Body Mass Index*				
< 18.5	2706 (15.9)	12 (0.3)	1500 (19.7)	1194 (20.2)
18.5–25	7766 (45.5)	115 (3.3)	3951 (51.8)	3700 (62.7)
> 25	1910 (11.2)	66 (1.9)	1400 (18.4)	444 (7.5)
Missing/unknown	4681 (27.4)	3340 (94.5)	776 (10.2)	561 (9.5)
Presence of moderate or severe anemia*				
No	6496 (38.1)	1177 (33.3)	2768 (36.3)	2551 (43.2)
Yes	3662 (21.5)	437 (12.4)	2022 (26.5)	1203 (20.4)
Missing/unknown	6901 (40.5)	1919 (54.3)	2837 (37.2)	2145 (36.4)
Accompagnateur named*				
No	12,792 (75.0)	3093 (87.5)	5540 (72.6)	4159 (70.5)
Yes	4267 (25.0)	440 (12.5)	2087 (27.4)	1740 (29.5)
Counseling prior to ART start*				
No	10,970 (64.3)	3065 (86.8)	4407 (57.8)	3498 (59.3)
Yes	6089 (35.7)	468 (13.2)	3220 (42.2)	2401 (40.7)
TB treatment or prophylaxis at baseline*				
No	9872 (57.9)	2534 (71.7)	4218 (55.3)	3120 (52.9)
Yes	7187 (42.1)	999 (28.3)	3409 (44.7)	2779 (47.1)
Cotrimoxazole prophylaxis at baseline*				
No	1470 (8.6)	1058 (29.9)	277 (3.6)	135 (2.3)
Yes	15,589 (91.4)	2475 (70.1)	7350 (96.4)	5764 (97.7)

Notes: Chi-square test for heterogeneity: *p < 0.0001.

Finally, we performed several sensitivity analyses to test the assumptions in our exploratory analysis of attrition and its associated factors. First, we performed the analysis using an alternative definition of the attrition outcome, based upon having more than 180 days elapse following a clinical visit [20]. Next, we considered the Option B+ group to include all women starting ART within 6 months (rather than 3 months) post-partum. Then, to assess the impact of a high degree of missing data in certain covariates, we performed an analysis using our primary outcome while dropping highly missing covariates (BMI, presence of moderate or severe anemia, household size, and presence of an HIV-positive household member) from the analysis. Last, to address violations of the proportional hazards assumption, we performed sensitivity analyses which were further stratified on particular covariates. Stata 13.1 (Stata Corp, College Station, TX) was used for all analyses.

### Ethical review

The study received scientific and ethical review and approval from the US Centers for Disease Control and Prevention and the Haiti National Committee on Bioethics.

## Results

A total of 17,059 patients from 73 health facilities representing nearly 50% of new ART patients from October 2012 to August 2014 were included in the analyses (Supplemental Digital Content 1). Similar to the distribution of health facilities in the country, over one-third of facilities were in the West Department, where the capital, Port-au-Prince, is located (Supplemental Digital Content 2: Facility Characteristics).

The patient cohort included 3533 Option B+ clients (20.7%), 7627 non-pregnant/non-breastfeeding women (44.7%), and 5899 men (34.6%) (Table 1). Of all patients, 51.5% were married, 60.7% lived in the same commune (district) as the health facility, 74.4% received the first-line ART regimen of tenofovir + lamivudine + efavirenz (TDF+3TC+EFV), and 91.4% received Cotrimoxazole prophylaxis prior to initiating ART. There were important differences across the three patient groups: Option B+ clients are younger with 82.1% aged 35 years old or younger compared to 41.7% for non-pregnant/non-breastfeeding women and 33.5% for men. Sixty-six percent of Option B+ clients were married or partnered compared to 45.6% and 50.0% for non-pregnant/non-breastfeeding women and men, respectively. Option B+ clients had less advanced HIV disease by WHO clinical staging or baseline CD4 (Table 1). Consistent with the need to start treatment rapidly to prevent MTCT, they were more likely to start ART rapidly, within seven days of enrollment in HIV care, and were less likely to have an *accompagnateur* named, to have pre-ART counseling, or to be receiving treatment or prophylaxis for tuberculosis or other opportunistic infections (OI) (Table 1).

**Figure 1.**
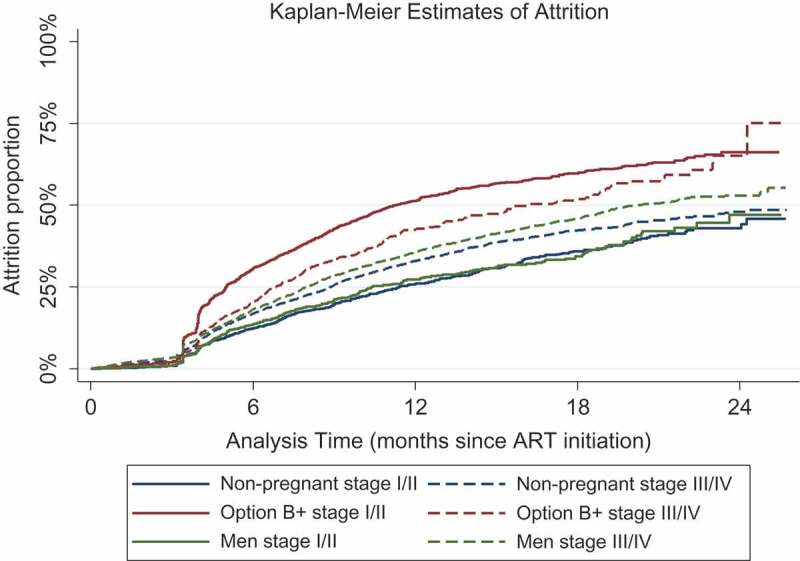
ART attrition by time since ART initiation by patient group and HIV disease stage.

The median follow-up time for patients in the study was 270 days (inter-quartile range [IQR]: 167–458 days). The Kaplan–Meier estimate of attrition was 50.4% by 12 months among Option B+ cases, 31.8% among non-pregnant/non-breastfeeding women, and 34.5% among men. Figure 1 demonstrates attrition rates over time by patient group and by level of HIV disease progression. A few patients were officially discontinued from ART prior to 90 days. Among non-pregnant/non-breastfeeding women and men, attrition was higher among those with more advanced HIV disease, but among Option B+ clients the opposite was observed. At 12 months, attrition was 25.9% and 27.3% among non-pregnant/non-breastfeeding women and men, respectively, with stage I/II disease but 32.9% and 35.7% among those with stage III/IV disease. In contrast, for Option B+ clients, attrition was 51.2% among those with WHO stage I/II vs 42.5% with WHO stage III/IV disease (Figure 1). Average rates of attrition are shown in Supplemental Digital Content 3. The average rate was higher among Option B+ clients (62.5 per 100 person years (PY)) than among other patients (35.2 per 100 PY for non-pregnant/non-breastfeeding women and 39.2 per 100 PY for men). Average rates were also notably higher among patients who were younger and who started ART rapidly (Supplemental Digital Content 3).

The elevated risk of attrition among Option B+ clients persisted after adjustment for covariates in the Cox regression model (Table 2). The association between HIV disease progression and attrition was modified by patient group (*p* < 0.001 for joint Wald test of the interaction terms). Option B+ clients classified as WHO stage I or II had the highest excess risk (59%) compared to the reference group (other women of WHO stage I or II). In contrast, among non-pregnant/non-breastfeeding women and men, attrition risk was elevated among those classified as WHO stage III or IV.

**Table 2.** Adjusted analysis of risk for ART attrition among ART patients in Haiti (n = 17,059).

	Hazard ratio	95% confidence interval	*p*-value
Patient group (reference = non-pregnant/breastfeeding women, WHO stage I or II)^a^			
Non-pregnant/breastfeeding women, WHO stage III or IV	1.14	(1.03, 1.26)	0.013
Option B+, WHO stage I or II	1.59	(1.42, 1.79)	0.000
Option B+, WHO stage III or IV	1.31	(1.13, 1.51)	0.000
Men, WHO stage I or II	1.11	(0.96, 1.29)	0.153
Men, WHO stage III or IV	1.35	(1.22, 1.50)	0.000
Marital status (reference = married/partnered)			
Widowed/divorced	1.03	(0.95, 1.12)	0.479
Single	0.94	(0.87, 1.01)	0.101
Missing/unknown	1.00	(0.93, 1.08)	0.924
Age^a^			
Each 10-year increase	0.98	(0.98, 0.99)	0.0001
Residence in same commune as health facility (reference = no)^c^			
Yes	0.94	(0.88, 1.00)	0.042
Household size (reference = 1–3 members)			
4+ members	1.01	(0.93, 1.11)	0.787
Any known HIV+ household members (reference = no)^a^			
Yes	0.79	(0.69, 0.90)	0.0001
Period of ART initiation (reference = Oct12–Mar13)^a^			
Apr13–Sep13	1.18	(1.10, 1.26)	0.0001
Oct13–Mar14	1.17	(1.08, 1.26)	0.0001
Apr14–Sep14	1.11	(0.99, 1.25)	0.079
ART starting regimen (reference = TDF+3TC+EFV)^a^			
AZT-3TC-EFV	1.03	(0.93, 1.14)	0.546
AZT-3TC-NVP	0.95	(0.87, 1.03)	0.216
TDF-3TC-NVP	1.50	(1.30, 1.72)	0.0001
All other	1.30	(1.07, 1.59)	0.008
Starting ART within 7 days of enrollment in care (reference = no)^a^			
Yes	1.41	(1.32, 1.51)	0.0001
Baseline BMI^a^			
Each 1-unit increase	0.97	(0.96, 0.98)	0.0001
Anemia (reference = normal or mild anemia)^a^			
Moderate or severe anemia	1.32	(1.23, 1.42)	0.0001
Treatment buddy named (reference = no)			
Yes	0.94	(0.87, 1.02)	0.120
Pre-ART counseling provided (reference = no)^a^			
Yes	0.84	(0.78, 0.91)	0.0001
TB-related treatment or prophylaxis at baseline (reference = no)			
Yes	0.94	(0.89, 1.00)	0.059
Cotrimoxazole prophylaxis at baseline (reference = no)^b^			
Yes	0.88	(0.80, 0.97)	0.007

Notes: Wald test: ^a^*p *< 0.0001, ^b^*p* < 0.01, ^c^*p* < 0.05.

After adjustment for all covariates in the stratified Cox model, we found an elevated risk of attrition associated with rapidly starting ART after enrollment in HIV care (*p* < 0.0001) and with having moderate or severe anemia (*p* < 0.0001). Patients who initiated ART with the regimen tenofovir + lamivudine + nevirapine (TDF+3TC+NVP) (*p* < 0.001) or other non-standard regimens (*p* < 0.01) were at elevated risk of attrition compared to those who started on the most common first-line regimen (TDF+3TC+EFV). Key protective factors were older age (*p* < 0.0001), living in the same commune as the health facility (*p* = 0.04), having another known HIV-positive household member (*p* < 0.0001), having greater BMI (*p* < 0.0001), having pre-ART counseling (*p *< 0.0001), and having received Cotrimoxazole prophylaxis for OI during baseline (*p* < 0.01).

In the sensitivity analysis using the alternative definition of ART attrition (more than 180 days since last visit), we found a lower absolute estimate of attrition (29.1% vs. 36.7% at 12 months in the sensitivity vs. the primary analysis), but with a similar, highly significant pattern of disparity between Option B+ clients and other patients. Findings on risk factors and protective factors were similar in magnitude and direction to the main analysis. Only 30 women were reclassified as Option B+ clients using the 6 months cutoff for breastfeeding, and the change had no significant impact upon our results. Our analysis which dropped covariates with highly missing data also yielded hazard ratio estimates which were consistent in direction and largely consistent in magnitude (with overlap in all 95% confidence intervals). The test for proportional hazards for our primary model yielded evidence of violation of the assumption for the variables rapidly starting ART after enrollment in care as well as presence of moderate or severe anemia. The estimates should therefore be treated as average hazard ratio estimates over time.

The assessed silent transfers represented only a small minority of attrition cases. Of 6249 patients classified as attrition cases in the survival analysis, it was possible to match 5904 of these cases (94.5%) within the HASS database. There were 418 cases (7.1%) with an alternative record for the same unique individual identified. Silent transfers among attrition cases were slightly more prominent among Option B+ clients (143/1766, or 8.1%) than among non-pregnant/breastfeeding women (151/2440, or 6.2%) or among men (124/2043, or 6.1%).

## Discussion

This is the first study on ART attrition in Haiti to specifically examine ART attrition among Option B+ clients relative to other ART clients. The overall attrition level in our study – 36.7% attrition at 12 months among all clients – was higher than those reported in the early days of ART programs in Haiti [10,12]. Most striking was the markedly elevated level of attrition among Option B+ clients, 50.4% at 12 months, compared with other adults. Our primary analysis considered cumulative incidence of attrition, which often reflects clinically meaningful interruption in ART. Our finding of excess risk of attrition among Option B+ clients was robust to alternative definitions of attrition. In a country like Haiti where HIV viral load is not routinely available, the incidence of attrition is a relevant programmatic indicator of patient clinical outcomes including viral load suppression and survival [7].

By linking iSanté data to data in HASS, we were able to investigate the potential role of silent transfers in this population with a relatively high level of attrition in general and in particular among pregnant women. Only 7.1% of clients had a duplicate record, indicating engagement in healthcare at a different facility after attrition was evident in iSanté. Though silent transfers were slightly more prevalent among the Option B+ group at 8.1%, overall this phenomenon explained only a small portion of the attrition observed across all groups. Silent transfers are relatively low in our study compared to other findings in East Africa. For example, a study across several sites in different East African countries found proportions of up to 17% for silent transfers among LTFU patients over a similar period of 2 years [17]. These findings have important implications for Haiti’s national HIV response, as well as for other LMICs scaling-up Option B+ and WHO’s recommended ‘test and start’ guidelines.

Other countries have also reported elevated attrition with Option B+ [21,22]. Fair comparisons between absolute attrition levels reported across studies are difficult to make given the wide heterogeneity of attrition definitions used in various studies, the sensitivity of estimates to the particular definition used, and the variety of methods for ascertaining attrition [8,20,23–26]. In Malawi, attrition at 6 months was 23.9% among Option B+ clients vs. 9.6% among women who initiated ART because of low CD4 counts or advanced disease stage [22]; these are lower attrition rates in absolute terms than those found in our study, but with a consistent pattern of disparity.

An important finding of our study was the relationship of attrition to stage of HIV disease progression at the time of ART initiation. Whereas more advanced disease stage was a risk factor for attrition among non-pregnant women and men, early HIV disease stage was a risk factor for Option B+ clients. Consistent with other studies, advanced HIV at ART initiation is associated with a higher proportion of adverse outcomes including death [25,27]. However, the lack of documentation of death among HIV patients due to a weak vital registration system in the country limits the capacity to disaggregate cases of attrition which in turn tends to inflate the proportion of LTFU. The association between early stage of HIV disease at ART initiation and attrition among pregnant women has been reported elsewhere; in a cohort followed by Giuliano et al. early stage of disease (CD4 > 350) was the main factor associated with LTFU between 6 and 24 months [21]. Another significant finding is the association between rapid initiation of ART after HIV diagnosis and elevated risk of attrition. The absence of or inadequate pre-ART adherence counseling before enrollment may be the explanation [15]. However, a study conducted at the GHESKIO Centers in Haiti has shown higher retention among the same-day ART initiation group than the standard ART initiation group [28]. Additional attrition risk factors include younger age [21,29] and moderate or severe anemia [21].

Protective factors in our study include living in the same commune as the health facility, pre-ART adherence counseling, having another known HIV-positive household member, and having received Cotrimoxazole prophylaxis for opportunistic infections at baseline; these were consistent with other studies [14,30].

The high risk of attrition under Option B+ represents a significant challenge and argues for targeted interventions to reinforce linkage to care and adherence and strengthen retention of pregnant women in order to reduce the potential impact on vertical transmission and improve health outcomes of HIV-infected mothers. Several studies have described successful interventions to improve retention in care and treatment services [31–33], including food assistance programs [33,34], the use of mobile phone technologies [35], accompagnateurs [12], and community-based support [13,36]. However, given the scarcity of resources and the increasing number of patients in need of ART, scaling up these interventions or sustaining them over time is a major challenge. ART programs in Haiti and other LMICs may need to rely more on community-based approaches that leverage patients’ own resources and capitalize on existing networks of social relationships to overcome barriers [37]. The community ART group approach implemented in Mozambique has shown promising results among stable non-pregnant patients [38]; a modified version was successfully implemented in the south of Haiti [39]. In the context of Option B+ and ‘test and start’, the question of long-term adherence on ART is even more relevant. There is an urgent need to identify determinants of long-term adherence and to design strategies and program interventions to help people living with HIV who are otherwise healthy achieve and maintain high levels of adherence to treatment.

## Limitations

There are several limitations to the iSanté EMR which may have affected our findings. First, our imputation of start and end dates of pregnancy among a large portion of patients may have also resulted in misclassification of Option B+ cases. Second, iSanté lacks information on some ART retention-related factors, such as education, socio-economic status, and prior disclosure of HIV status to others. Third, certain variables had high levels of missing data, particularly on household composition and baseline BMI and anemia. Our use of multiple imputation assumed these data were missing completely at random or that the missingness could be explained by observed covariates, both of which are untestable assumptions, and unpredictable bias may be present in our estimates. However, our sensitivity analysis provided some reassurance of stability in our estimates of excess risk. Fourth, our sensitivity analyses found that the risk associated with rapidly starting ART was not proportional over time and was variable by patient group. More work is needed to explore the particular relationship between timing of ART start relative to testing and enrollment in care and risk of attrition, in combination with patient group and other predictors. Finally, the exclusion of 31% of clinics using iSanté due to delay in transferring data to the central server may have biased our results and limited their generalizability. Despite these limitations, the iSanté EMR represents a valuable source of information for exploring patterns of ART attrition in a large cohort of Haitian patients.

## Conclusion

Overall attrition from ART services among pregnant and non-pregnant patients following adoption of Option B+ in Haiti is high. Option B+ clients initiating ART at an early stage of HIV disease present the highest risk for attrition. Tailored interventions ensuring consistent pre-ART adherence counseling, promoting disclosure and high-quality family-centered care, reducing distances between services delivery and beneficiaries, and capitalizing on existing networks of social relationships could help reduce attrition within Haiti’s national ART program.

## Supplementary Material

Supplemental Digital Content 3

Supplemental Digital Content 2

Supplemental Digital Content 1

## Paper context

Early results on HIV patient retention are promising; however, levels of retention vary. In Haiti, programs exist to promote patient retention, which is observed as moderate to high. After over two years of Option B+ and use of the 2013 WHO treatment guidelines, few studies have explored patient attrition. None have looked at pregnant women. This study examines the levels of and factors associated with attrition in pregnant and non-pregnant adults initiating ART in Haiti.
