# Supplementary material for: Attrition from antiretroviral treatment services among pregnant and non-pregnant patients following adoption of Option B+ in Haiti
Source: Glob Health Action. 2017 Jun 22;10(1):1330915. doi: 10.1080/16549716.2017.1330915 (PMC5496080; doi:10.1080/16549716.2017.1330915)
Supplement: Supplemental Digital Content 3 [file zgha_a_1330915_sm1794.pdf]

Supplemental Digital Content 3: Average Attrition Rates by Covariates

	Attrition rate per 100 PY	95% Confidence Interval
All patients	41.7	(40.7, 42.8)
Patient group [a]		
Non-pregnant females	35.2	(33.8, 36.6)
Pregnant/Breastfeeding females	62.5	(59.6, 65.4)
Males	39.2	(37.5, 40.9)
Marital status [b]		
Married/partnered	42.8	(41.3, 44.3)
Widowed/divorced	37.2	(34.6, 39.9)
Single	42.7	(40.2, 45.3)
Missing/Unknown	41.3	(39.0, 43.8)
Age group [a]		
<25 years	67.3	(63.5, 71.3)
25-35 years	46.1	(44.2, 48.0)
35-50 years	35.1	(33.6, 36.6)
>50 years	31.0	(28.9, 33.2)
Location of residence relative to clinic [a]		
Different commune	39.2	(37.6, 40.8)
Same commune	43.3	(42.0, 44.7)
Missing/Unknown	42.4	(35.6, 50.5)
Household size [a]		
1-3 members	37.5	(35.8, 39.2)
4+ members	33.4	(30.8, 36.3)
Missing/Unknown	45.9	(44.5, 47.4)
Other known HIV+ person in household [a]		
No	38.2	(36.6, 39.9)
Yes	27.7	(24.8, 31.0)
Missing/Unknown	45.9	(44.5, 47.4)
Timing of ART start [a]		
Oct12-Mar13	37.9	(36.3, 39.6)
Apr13-Sep13	45.9	(44.1, 47.7)
Oct13-Mar14	44.8	(42.5, 47.3)
Apr14-Sep14	33.0	(29.9, 36.3)
Starting ART regimen [a]		
TDF-3TC-EFV	42.0	(40.8, 43.2)
AZT-3TC-EFV	35.5	(33.2, 37.9)
AZT-3TC-NVP	45.2	(41.5, 49.1)
TDF-3TC-NVP	53.3	(47.0, 60.4)
All Other	57.4	(47.9, 68.8)
ART start within 7 days of enrollment [a]		
No	35.5	(34.5, 36.6)

Yes	62.9	(60.3, 65.6)
WHO stage at baseline (by staging or CD4) [a]		
I	51.9	(49.1, 55.0)
II	34.6	(32.3, 37.0)
III	36.1	(34.6, 37.7)
IV	44.0	(41.9, 46.1)
Missing/Unknown	64.8	(59.4, 70.7)
Baseline body mass index [a]		
<18.5	46.4	(43.7, 49.3)
18.5-25	32.5	(31.3, 33.9)
>25	22.9	(20.8, 25.2)
Missing/Unknown	66.2	(63.6, 68.9)
Presence of moderate or severe anemia [a]		
No	31.0	(29.7, 32.5)
Yes	45.5	(43.3, 47.9)
Missing/Unknown	51.5	(49.6, 53.4)
Treatment supporter named [a]		
No	45.1	(43.8, 46.3)
Yes	32.5	(30.8, 34.4)
Counseling prior to ART start [a]		
No	49.9	(48.5, 51.4)
Yes	28.4	(27.0, 29.8)
TB treatment or prophylaxis at baseline [a]		
No	43.6	(42.2, 45.0)
Yes	39.0	(37.5, 40.6)
Cotrimoxizole prophylaxis at baseline [a]		
No	68.4	(63.8, 73.3)
Yes	39.5	(38.4, 40.5)

Log rank test for equality of survivor function: [b] p<0.001, [a] p<0.0001
