# Supplementary material for: Attrition from antiretroviral treatment services among pregnant and non-pregnant patients following adoption of Option B+ in Haiti
Source: Glob Health Action. 2017 Jun 22;10(1):1330915. doi: 10.1080/16549716.2017.1330915 (PMC5496080; doi:10.1080/16549716.2017.1330915)
Supplement: Supplemental Digital Content 2 [file zgha_a_1330915_sm1793.pdf]

Supplemental Digital Content 2: Facility Characteristics (n=73)

	N	%
Total	73	100.0
Location (Department)		
Artibonite	6	8.2
Grand'Anse	5	6.8
Nippes	2	2.7
Nord	13	17.8
Nord Est	6	8.2
Nord Ouest	3	4.1
Ouest	26	35.6
Sud	8	11.0
Sud Est	4	5.5
Category		
Government/public	36	49.3
Mission/Faith-based	17	23.3
NGO/Private not for profit	8	11.0
Private for profit	11	15.1
Missing/Unknown	1	1.4
Type		
Dispensary	5	6.8
Health Center without beds	21	28.8
Health Center with beds	10	13.7
Hospital / Community reference hospital	26	35.6
Departmental / University hospital	10	13.7
Missing / Unknown	1	1.4
