# Supplementary figures and images for: Attrition from antiretroviral treatment services among pregnant and non-pregnant patients following adoption of Option B+ in Haiti
Source: Glob Health Action. 2017 Jun 22;10(1):1330915. doi: 10.1080/16549716.2017.1330915 (PMC5496080; doi:10.1080/16549716.2017.1330915)

## Supplemental Digital Content 1: Study Population

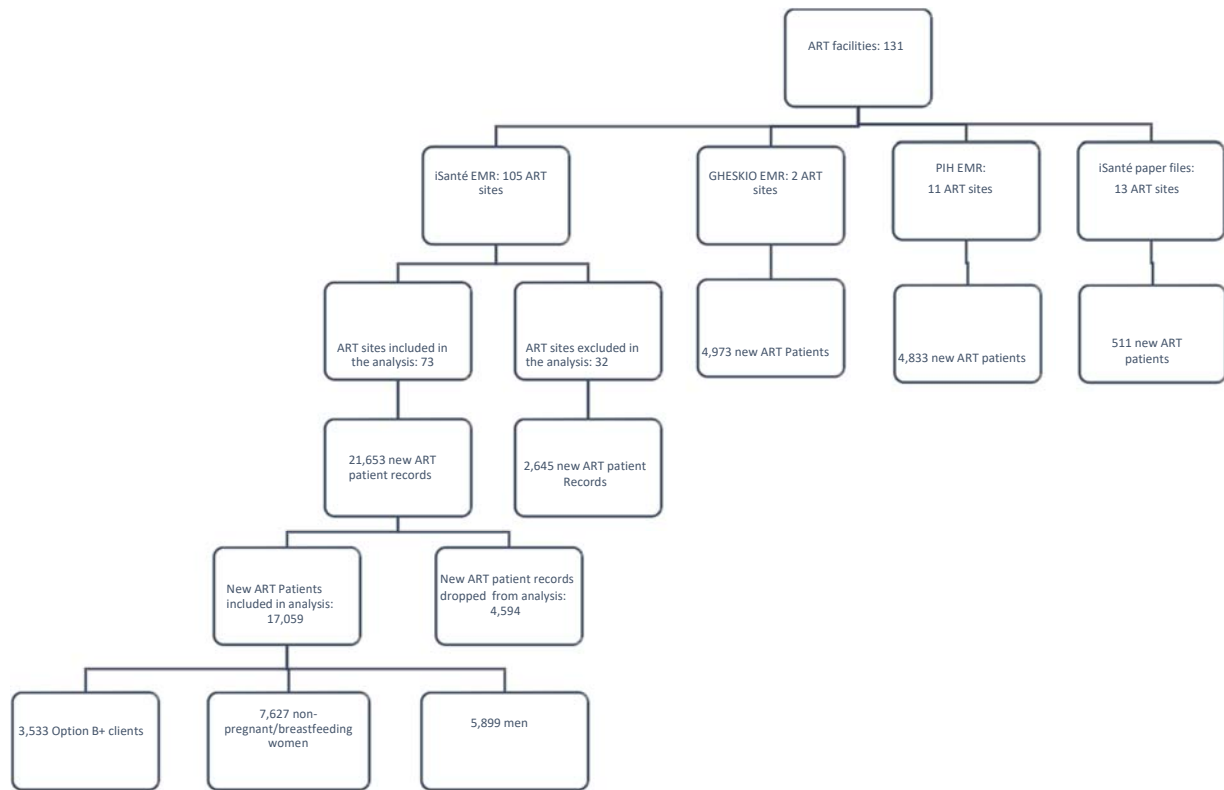

Supplement: Supplemental Digital Content 1 [file zgha_a_1330915_sm1787.pdf]
